# The Guest Hospital, Dudley

**Published:** 1920-05-29

**Authors:** Arthur Bird

**Affiliations:** Secretary. The Guest Hospital, Dudley


### THE EDITOR'S LETTER-BOX.

**Correspondence on all subjects is invited, but we cannot in any way be responsible for the opinions expressed by our correspondents, who must give their name and address as a guarantee of good faith, but not necessarily for publication. Correspondents are reminded that brevity of style and conciseness of statement greatly facilitate early insertion.**

### The Guest Hospital, Dudley.

*To the Editor of* The Hospital.

Sir,—Will you kindly allow me to say a few words with respect to your article on the Guest Hospital in this week's Hospital?

The correspondence between Sir George Bean and myself took place more than two years ago, but even before then the best *x*-ray electrical apparatus that money could buy had been installed, and it has always been efficiently worked. Apart from experts, it would be difficult to find a more efficient operator than our senior R.M.O., Mr. Butterworth, who has been responsible for the work during the last two years.

Sir George Bean represents all that is best in the public life of Dudley. He is interested in its progress and welfare, and I am hoping that my efforts to secure his influence in favour of this hospital will be successful.—Yours faithfully, Arthur Bird,

The Guest Hospital, Dudley, Secretary.

May 21, 1920.

